# Living longer and lifestyle: A report on the oldest of the old in the Adventist Health Study-2

**DOI:** 10.1016/j.jarlif.2025.100010

**Published:** 2025-04-11

**Authors:** Hildemar Dos Santos, Alaa Alabadi-Bierman, Michael Paalani, Sen Luu Padilla, Abel Alvarez, W. Lawrence Beeson, Gary E. Fraser

**Affiliations:** a School of Public Health, Loma Linda University, 24951 North Circle Drive, Nichol Hall, Room 1306, Loma Linda, CA 92350, United States; b Adventist Health Study, Research Affairs, Loma Linda University, Loma Linda, CA, United States; c Center for Nutrition, Healthy Lifestyles and Disease Prevention, School of Public Health, Loma Linda University, Loma Linda, CA, United States; d Department of Medicine, School of Medicine, Loma Linda University, Loma Linda, CA, United States

**Keywords:** Longevity, Older adult, Aging, Vegetarianism, Adventist health

## Abstract

**Objective:**

This investigation aimed to evaluate and describe the health profile and dietary patterns of the oldest Adventists (individuals aged 80 years and older).

**Design:**

Cross-sectional investigation.

**Setting:**

Self-administered lifestyle questionnaire in Adventist congregations in North America.

**Participants:**

7192 individuals aged 80 years of age or older enrolled in the Adventist Health Study-2.

**Measurements:**

Dietary intakes for participants were evaluated using a self-administered quantitative food frequency questionnaire. Selected health outcomes data were assessed with the baseline self-administered medical history questionnaire.

**Results:**

Our cohort of the old adults Adventists had a predominant female participation (62 %), and the percentage of vegetarians was 52.7 %. Based on classification into respective dietary patterns, 7.8 % of the study population were vegan, 29.2 % of the participants were lacto-ovo vegetarians, 10.2 % were pesco-vegetarians, 5.5 % were semi-vegetarians, and 47.3 % were non-vegetarians. Regarding the assessment of prevalent conditions, non-vegetarians were more likely to report having hypertension than other dietary patterns. Semi-vegetarians and non-vegetarians were more likely to report high cholesterol. A large number of participants reported never smoking (78.5 %) and never drinking alcoholic beverages (57.8 %), and non-vegetarians reported the poorest health perception (20 %) compared to vegans (11.4 %).

**Conclusion:**

Our Adventist Health oldest of the old cohort shared many of the characteristics observed among the individuals that make up the long-living cohorts worldwide as well as younger aged Adventist participants. This observation indicates the importance of non-smoking, abstinence from alcohol consumption, daily engagement in regular physical activity, avoidance of disease in older ages, and following a plant-based diet concerning the potential for successful aging.

## 1 Introduction

Older adults represent one of the most rapidly expanding demographics as the United States (U.S.) population experiences improved aging and increased life expectancy [1]. As of 2022, the U.S. population aged 65 and older reached 57.8 million, accounting for 17.3 % of the total population. This marks a notable demographic change, with the older adult population increasing by 34 % over the past decade, rising from 43.1 million in 2012 to 57.8 million in 2022 [1]. Almost every state in the nation experienced an increase in median age [2]. This notable point has set the course for the nation to become older. According to the 2023 Profile of Older Americans, the population aged 85 and older is expected to more than double, increasing from 6.5 million in 2022 to 13.7 million by 2040—a 111 % increase [1].

Medical advancements, better sanitation, changes in lifestyle, and a larger proportion of adults, partly due to the baby boom after the end of World War II, are some factors contributing to the expansion of the older adult population in the U.S [3]. However, with an older population, increases in disability and decreases in health yield challenges to our healthcare system and clinical practices [4,5].

The determinants of longevity are unclear because risk factors for dying change as our age increases. There are some areas around the world where people live longer, termed “Blue Zones [4].” These areas are Sardinia (Italy) [6], Okinawa (Japan) [7], Loma Linda, California (U.S.) [8], Nicoya (Costa Rica), and Ikaria (Greece) [9]. Several global studies have reported that individuals residing in Blue Zone regions exhibit notably high life expectancy [[6], [7], [8], [9]] and are ten times more likely to live to 100 years of age [4]. Individuals living in Blue Zones have been successful at avoiding disease and/or at least delaying death. From the search for factors related to longevity in people living in Blue Zones, it has been purported there are key lifestyle and behavioral patterns. Some common lifestyle characteristics observed among these individuals included family engagement, social engagement, less smoking, moderate alcohol intake, regular physical activity, and a plant-based diet [10]. Among the five Blue Zone areas, Loma Linda is the only area that overtly and intensively promotes a plant-based dietary intake pattern [4,8,11].

The Adventist Health Studies (AHS) are a series of long-term research projects that began in the late 1950s to explore the health impacts of diet and lifestyle in the Seventh-day Adventist (SDA) community. Known for their emphasis on vegetarianism, regular physical activity, and avoidance of alcohol, tobacco, and caffeine, Adventists provide a unique cohort for studying how lifestyle factors contribute to health outcomes and longevity. The first cohort, The Adventist Mortality Study (AMS) (1958–1966) was succeeded by the Adventist Health Study-1 (AHS-1), which followed >34,000 participants, and subsequent studies, such as Adventist Health Study-2 (AHS-2), have expanded the sample size to include tens of thousands of individuals. The significance of the AHS lies in its ability to isolate the effects of lifestyle choices on chronic diseases, with findings showing a strong correlation between plant-based diets and reduced risk of heart disease, cancer, and diabetes [8]. These studies have positioned the AHS as a key source of evidence in the broader field of longevity research, providing valuable insights into the role of diet and health practices in promoting lifespan and quality of life [9]. As such, the Adventist Health Studies have contributed to global public health initiatives and are often cited in research advocating for dietary and lifestyle changes to improve long-term health outcomes.

Most studies examining older adults have concentrated on specific communities within distinct regions [[10], [11], [12]]. Additionally, other studies have investigated centenarians independently of the AHS. In contrast, our study aimed to assess a broader sample of centenarians across the U.S. based on faith (i.e., members of the Adventist church), encompassing diverse occupations, geographic locations, and cultural backgrounds. Our objectives for this study were threefold: 1) provide a description of the older adult Adventist population regarding the dietary intake and specific lifestyle patterns, 2) determine what characteristics, considered to be important for longevity, are shared between our sample and other long-living cohorts, and 3) cross-sectionally evaluate associations among diet pattern and certain chronic conditions (such as hypertension, high cholesterol, type-2 diabetes, angina, stroke, transient ischemic attack, congestive heart failure, and cancer).

## 2 Material and methods

### 2.1 Study sample

Our sample was obtained from the Adventist Health Study-2 (AHS-2) archival data. The AHS-2 is a large prospective North American cohort study with over 96,000 participants, and recruitment targeted English-speaking members of the Seventh-day Adventist Church in Canada and the U.S. from 2002 to 2007. Participants completed a self-administered lifestyle questionnaire regarding diet, including consumption of vegetarian products, physical activity, supplement use, and medical history [13]. The number of participants included in the study was based on the following exclusion criteria: missing data for birth date, sex, race, or questionnaire return date, and current smokers because there were only a few current smoking participants. Additionally, anyone younger than 80 was also excluded at the time of enrollment into the AHS-2. The remaining analytic sample consisted of 7192 individuals.

### 2.2 Measurements

*Dietary Data*: Participants' dietary intakes were evaluated using a self-administered quantitative food frequency questionnaire (FFQ) comprising over 200 food items. Specification for dietary patterns was based on reported consumption of animal protein. Hence, vegans reported consumption of animal protein less than one time per month; lacto-ovo vegetarians reported consumption of eggs and dairy equal to or greater than one time per month, but all other animal products less than one time per month; pesco-vegetarians reported consuming fish one time per month or more, and all other meats less than one time per month; semi-vegetarians reported consuming all combined meats one time per month or more but no more than one time per week, and their reported consumption of non-fish meats was equal to or greater than one time per month; and lastly, non-vegetarians reported consuming all combined meats greater than one time per week and non-fish meats one time per month or more.

*Selected Health Outcomes Data*: was assessed with the baseline self-administered medical history questionnaire. Disease status was determined according to self-report of disease. Thus, participants specified “Yes” to the question “Have you been treated for this …” (example: diabetes mellitus Type II adult onset) or provided one selection indicating the years since first diagnosis for the question “Years since diagnosis with…”.

*Covariates*: included in our study are the following demographic variables: age (as age in years at baseline), body mass index (BMI) (as kg/m^2^ at baseline), sex (dichotomous, as male/female), race/ethnicity (dichotomous, as Black/non-Black), education (3 levels, as high school or less, some college, and bachelor's or higher), and personal income (4 levels, as <$20,000, $21,000–50,000, $51,000–100,000, and ≥ $101,000).

In addition, the following lifestyle variables were included: exercise (5 levels, as none, ≤20 min/week, 21–60 min/week, 61–150 min/week, and ≥151 min/week), smoking (dichotomous, as never smoker/past smoker), alcohol intake (dichotomous, as never/ever), television watching (3 levels, as <1 hr/day, 1–2 hrs/day, and ≥3 hrs/day), sleep (3 levels, as <1 hr/night, 1–2 hrs/night, and ≥3 hrs/night), napping (3 levels, as <1 hr/day, 1–2 hrs/day, and ≥3 hrs/day) and hypertension (dichotomous, as yes/no).

### 2.3 Statistical analysis

For the descriptive analysis, the differences in covariate values by gender and dietary intake pattern were evaluated by Pearson's chi-square tests, and ANOVA/*t*-tests for categorical and continuous variables, respectively. Guided multiple imputations were used for missing dietary data [14]. A *p*-value of <0.05 was considered statistically significant. SAS version 9.4 (SAS Institute, Inc., Cary, NC) was used for all calculations.

## 3 Results

In Table 1, the socio-demographics, lifestyles, and clinical characteristics of the study population are presented. Overall, there are more female participants compared to males. The average age for males and females was 84.9 years. Among females, 8 % reported being vegan, 28.5 % reported lacto-ovo vegetarian intake pattern, 10.4 % reported pesco-vegetarian intake, 5.8 % reported semi-vegetarian pattern, and 47.3 % reported a non-vegetarian intake pattern. Among males, 7.5 % reported being vegan, 30.4 % reported lacto-ovo vegetarian intake, 9.9 % reported pesco-vegetarian intake, 4.9 % reported semi-vegetarian pattern, and 47.3 % reported non-vegetarian intake. There was a difference in BMI for males and females, with females having a higher BMI value of 27.2 compared to males with a value of 26.6. Other differences included females more often reporting never drinking alcohol, never smoking, having higher levels of education, napping, sleeping, having better-perceived health, and reporting lower yearly income than males. Females were also more likely to report having a diagnosis of hypertension but less frequent angina symptoms than males.

**Table 1:** Baseline Characteristics for AHS-2 Participants Aged 80+ at Enrollment (2002–2007).

Characteristics^a^ (*N* = 7192)	N ( %)		*P-value*
	Male	Female	
Participants	*N* = 2590 (36 %)	*N* = 4602 (64 %)	
Age, mean yr (SD)	84.9 (4.15)	84.9 (4.01)	
Dietary Pattern			0.28
Vegan	194 (7.49)	368 (8.00)	
Lacto-ovo vegetarian	787 (30.4)	1313 (28.5)	
Pesco-vegetarian	256 (9.88)	480 (10.4)	
Semi-vegetarian	128 (4.94)	266 (5.78)	
Non-vegetarian	1225 (47.3)	2175 (47.3)	
BMI, mean (SD)	26.6 (4.63)	27.2 (6.41)	**<0.0001**
BMI Categories (kg/m^2^)			**<0.0001**
<18.5	36 (1.4)	118 (2.6)	
18.5–24.9	969 (37.4)	1865 (40.5)	
25–29.9	1088 (42.0)	1373 (29.8)	
30+	497 (19.2)	1246 (27.1)	
Race			**<0.0001**
Black	514 (19.9)	1222 (26.6)	
Non-Black	2076 (80.2)	3380 (73.5)	
Personal Income, $1,000/year			**<0.0001**
≤20.0	433 (18.2)	1131 (33.5)	
20.1–50.0	1144 (48.1)	1645 (48.8)	
50.1–100.0	574 (24.2)	515 (15.3)	
≥100.1	226 (9.5)	81 (2.4)	
Alcohol consumption			**<0.0001**
Never	1358 (52.4)	2908 (63.2)	
Ever	1232 (47.6)	1694 (36.8)	
Educational Level			**<0.0001**
High School or less	491 (19.0)	986 (21.4)	
Some College	834 (32.2)	2014 (43.8)	
Bachelor's and above	1265 (48.8)	1602 (34.8)	
Smoking			**<0.0001**
Never	1907 (73.6)	3838 (83.4)	
Past Smoker	683 (26.4)	764 (16.6)	
Exercise, min/wk			**<0.0001**
None	455 (17.6)	1063 (23.1)	
≤20	507 (19.6)	911 (19.8)	
21–60	423 (16.3)	729 (15.8)	
61–150	624 (24.1)	1111 (24.1)	
≥151	581 (22.4)	788 (17.1)	
Nap, hrs/day			**<0.0001**
<1	1075 (41.5)	2215 (48.1)	
1–2	554 (21.4)	839 (18.2)	
≥3	961 (37.1)	1548 (33.6)	
Sleep, hrs/night			
≤4	60 (2.3)	116 (2.5)	
5–8	2387 (92.2)	4203 (91.3)	
≥9	143 (5.5)	283 (6.2)	
TV watching, hrs/day			**<0.0001**
<1	738 (28.5)	1154 (25.1)	
1–2	1219 (47.1)	2085 (45.3)	
≥3	633 (24.4)	1363 (29.6)	
Perceived Health			**<0.0001**
Excellent	809 (31.2)	995 (21.6)	
Good	1450 (56.0)	2791 (60.7)	
Fair/Poor	331 (12.8)	816 (17.7)	
High Blood Pressure			**<0.05**
No	1189 (72.9)	3230 (70.2)	
Yes	701 (27.1)	1372 (29.8)	
High Cholesterol			0.19
No	1933 (74.6)	3369 (73.2)	
Yes	657 (25.4)	1233 (26.8)	
Diabetes			0.76
No	2311 (89.2)	4117 (89.5)	
Yes	279 (10.8)	485 (10.5)	
Angina			**<0.05**
No	2499 (96.5)	4484 (97.4)	
Yes	91 (3.5)	118 (2.6)	
Stroke			0.20
No	2553 (98.6)	4552 (98.9)	
Yes	37 (1.4)	50 (1.1)	
Transient Ischemic Attack			0.51
No	2528 (97.1)	4480 (97.4)	
Yes	62 (2.4)	122 (2.7)	
Congestive Heart Failure			0.33
No	2544 (98.2)	4505 (97.9)	
Yes	46 (1.8)	97 (2.1)	
Cancer			0.99
No	2372 (91.6)	4215 (91.6)	
Yes	218 (8.4)	387 (8.4)	

a Data are frequency and percent unless otherwise indicated.

In Table 2, participants' characteristics were compared to their dietary patterns. The mean BMI was lowest for vegans and increased incrementally, with the highest BMI reported for non-vegetarians. Non-vegetarians were more likely to report ever smoking, ever drinking, and tended to have lower levels of physical activity. Black participants were more likely to be pesco-vegetarians and non-vegetarians. Non-vegetarians were most likely to report napping three or more hours per day, watching television three or more hours per day, having a lower educational level, and perceiving health as good or fair/poor. In addition, non-vegetarians were more likely to have BMI values in the overweight or obese categories.

**Table 2:** Baseline Characteristics for AHS-2 Participants Aged 80+ at Enrollment by Dietary Pattern (2002-2007).

	Dietary Pattern, N (%)					
Characteristics^a^^,^^b^	Vegetarian					*P-value*
	Vegan	Lacto-Ovo	Pesco	Semi	Non-vegetarian	
Participants	N=562 (7.8)	N=2100 (29.2)	N=736 (10.2)	N=394 (5.5)	N=3400 (47.3)	
Age, mean yr (SD)^c^	84.9 (4.0)	84.9 (4.2)	84.7 (3.9)	84.8 (3.9)	85 (4.0)	0.51
Sex						0.28
Male	194 (7.49)	787 (30.4)	256 (9.88)	128 (4.94)	1225 (47.3)	
Female	368 (8.00)	1313 (28.5)	480 (10.4)	266 (5.78)	2175 (47.3)	
BMI, mean (SD)^c^	23.9 (4.6)	25.7 (5.0)	26.0 (5.2)	27.2 (5.9)	28.5 (6.2)	**<.0001**
BMI Categories (kg/m^2^)						**<.0001**
<18.5	43 (7.7)	57 (2.7)	17 (2.3)	7 (1.8)	30 (0.9)	
18.5-24.9	328 (58.4)	1010 (48.1)	348 (47.3)	159 (40.4)	989 (29.1)	
25-29.9	133 (23.6)	694 (33.1)	241 (32.7)	124 (31.4)	1269 (37.3)	
30+	58 (10.3)	339 (16.1)	130 (17.7)	104 (26.4)	1112 (32.7)	
						
Race, Black	115 (20.5)	250 (11.9)	243 (33.0)	47 (11.9)	1081 (31.8)	**<.0001**
Personal Income, $1,000/year						0.10
≤20.0	289 (51.4)	889 (42.3)	288 (39.1)	188 (47.7)	1353 (40.0)	
20.1-50.0	188 (33.5)	798 (38.0)	298 (40.5)	137 (34.8)	1368 (40.2)	
50.1-100.0	63 (11.2)	326 (15.5)	119 (16.2)	49 (12.4)	532 (15.7)	
≥100.1	22 (3.9)	87 (4.1)	31 (4.2)	20 (5.1)	147 (4.3)	
Alcohol consumption						**<.0001**
Never	382 (68.0)	1541 (73.4)	477 (64.8)	253 (64.2)	1613 (47.4)	
Ever	180 (32.0)	559 (26.6)	259 (35.2)	141 (35.8)	1787 (52.6)	
Educational Level						**<.0001**
High School or less	107 (19.0)	302 (14.4)	147 (20.0)	91 (23.1)	830 (24.4)	
Some College	210 (37.4)	771 (36.7)	272 (37.0)	156 (39.6)	1439 (42.3)	
Bachelor's and above	245 (43.6)	1027 (48.9)	317 (43.0)	147 (37.3)	1131 (33.3)	
Smoking						**<.0001**
Never	469 (83.5)	1864 (88.8)	609 (82.7)	316 (80.2)	2487 (73.2)	
Ever	93 (16.5)	236 (11.2)	127 (17.3)	78 (19.8)	913 (26.8)	
Exercise, min/wk						**<.0001**
None	89 (15.8)	401 (19.0)	133 (18.1)	101 (25.6)	794 (23.4)	
≤20	92 (16.4)	400 (19.1)	119 (16.2)	81 (20.6)	726 (21.4)	
21-60	93 (16.6)	332 (15.8)	126 (17.1)	52 (13.2)	549 (16.2)	
61-150	143 (25.4)	547 (26.1)	188 (25.5)	83 (21.1)	774 (22.8)	
≥151	145 (25.8)	420 (20.0)	170 (23.1)	77 (19.5)	557 (16.4)	
Nap, hrs/day						**<.0001**
<1	330 (58.7)	1027 (48.9)	331 (45.0)	187 (47.5)	1415 (41.6)	
1-2	101 (18.0)	412 (19.6)	137 (18.6)	86 (21.8)	657 (19.3)	
≥3	131 (23.3)	661 (31.5)	268 (36.4)	121 (30.7)	1328 (39.1)	
Sleep, hrs/night						**<.0001**
≤4	11 (2.0)	20 (0.9)	27 (3.7)	7 (1.8)	111 (3.3)	
5-8	514 (91.5)	1951 (93.0)	679 (92.3)	367 (93.2)	3079 (90.6)	
≥9	37 (6.5)	129 (6.1)	30 (4.0)	20 (5.0)	210 (6.1)	
TV watching, hrs/day						**<.0001**
<1	283 (50.4)	715 (34.1)	206 (28.0)	100 (25.4)	588 (17.3)	
1-2	202 (35.9)	955 (45.5)	373 (50.8)	178 (45.2)	1596 (46.9)	
≥3	77 (13.7)	430 (20.5)	157 (21.3)	116 (29.4)	1216 (35.8)	
Perceived Health						**<.0001**
Excellent	188 (33.5)	656 (31.2)	191 (25.9)	109 (27.6)	660 (19.4)	
Good	310 (55.1)	1218 (58.0)	439 (59.7)	215 (54.7)	2059 (60.6)	
Fair/Poor	64 (11.4)	226 (10.8)	106 (14.4)	70 (17.7)	618 (20.0)	

a Data are frequency and percent unless otherwise indicated. Percentages might not total 100 because of rounding.

b Chi-square tests were used for analysis.

c ANOVA was used for the analysis.

In Table 3, the eight most prevalent health outcomes reported on the baseline enrollment questionnaire were evaluated to determine if there were any differences between dietary patterns. Non-vegetarians were more likely to report having hypertension (34 %) compared to all vegetarian groups. Vegans were the least likely to report hypertension (16.73 %). Semi-vegetarians and non-vegetarians were more likely to report high cholesterol (32.23 % and 29.76 %, respectively) compared to the other vegetarian groups. Vegans reported the lowest frequency of high cholesterol levels (16.73 %). Only 13.7 % of vegans reported watching three or more hours of TV daily compared to 35.8 % of non-vegetarians. For other selected health outcomes, there were no differences between the distinct dietary patterns.

**Table 3:** Select Health Outcomes for AHS-2 Participants Aged 80+ at Enrollment by Dietary Pattern (2002-2007).

Health Outcomes^a^^,^^b^	Dietary Pattern, N (%)					*P-value*
	Vegetarian					
	Vegan	Lacto-Ovo	Pesco	Semi	Nonvegetarian	
Hypertension						**<.0001**
No	468 (83.27)	1619 (77.10)	522 (70.92)	269 (68.27)	2241 (65.91)	
Yes	94 (16.73)	481 (22.90)	214 (29.08)	125 (31.73)	1159 (34.09)	
High Cholesterol						**<.0001**
No	470 (83.63)	1615 (76.90)	562 (76.36)	267 (67.77)	2388 (70.24)	
Yes	92 (16.37)	485 (23.10)	174 (23.64)	127 (32.23)	1012 (29.76)	
Diabetes type 2						0.99
No	500 (88.97)	1875 (89.29)	657 (89.27)	353 (89.59)	3043 (89.50)	
Yes	62 (11.03)	225 (10.71)	79 (10.73)	41 (10.41)	357 (10.50)	
Angina						0.19
No	553 (98.40)	2029 (96.62)	719 (97.69)	383 (97.21)	3299 (97.03)	
Yes	9 (1.60)	71 (3.38)	17 (2.31)	11 (2.79)	101 (2.97)	
Stroke						0.91
No	557 (99.11)	2076 (98.86)	726 (98.64)	390 (98.98)	3356 (98.71)	
Yes	5 (0.89)	24 (1.14)	10 (1.36)	4 (1.02)	44 (1.29)	
Transient Ischemic Attack						0.11
No	553 (98.40)	2045 (97.38)	724 (98.37)	379 (96.19)	3307 (97.26)	
Yes	9 (1.60)	55 (2.62)	12 (1.63)	15 (3.81)	93 (2.74)	
Congestive Heart Failure						0.50
No	554 (98.58)	2065 (98.33)	721 (97.96)	386 (97.97)	3323 (97.74)	
Yes	8 (1.42)	35 (1.67)	15 (2.04)	8 (2.03)	77 (2.26)	
Cancer						0.24
No	511 (90.93)	1903 (90.62)	677 (91.98)	358 (90.86)	3138 (92.29)	
Yes	51 (9.07)	197 (9.38)	59 (8.02)	36 (9.14)	262 (7.71)	

a Data are frequency and percent unless otherwise indicated.

b Chi-square tests were used for analysis.

## 4 Discussion

Our investigation reported the characteristics of the older adult Seventh-day Adventists (SDAs) participating in the AHS-2. Because of the large number of older adult participants and vegetarians in this cohort, we were able to determine participant dietary intake patterns, lifestyles, and demographic factors. In addition, we could evaluate if our participants share characteristics that contribute to longevity with other cohorts and populations. With this assessment, our observations provide a foundation for understanding which modifiable lifestyle factors are essential for longevity and how those factors may be related to specific morbidities. The cross-sectional nature of this study limits the ability to establish causal relationships. Therefore, while associations between lifestyle factors and outcomes can be identified, this study does not provide evidence that lifestyle directly caused these outcomes. Therefore, our findings do not support definitive conclusions regarding causation.

Similar to previous studies evaluating long-living populations, the participants in our investigation were mainly females. This observation agrees with prior investigations, like the Gerontology Research Group, where researchers observed that the longest-living individuals are typically females [[15], [16], [17], [18]]. Yet, it cannot be ignored that females may be more likely to complete and return a mailed lifestyle questionnaire than males, resulting in response bias [19]. Nevertheless, it has been noted that the life expectancy difference between men and women is a worldwide phenomenon where the longevity observed in females is created by a combination of biological characteristics as well as lifestyle, life experiences, behavior, and social roles. Aside from factors affecting women, elements affecting men have also contributed to women outliving men. For instance, health-related risk factors are more prevalent in males compared to females, such as smoking and poor dietary intake, which have played a role in excess male mortality [18,20]. Female participants in our study reported lower levels of “past smoking,” “drinking alcohol,” and angina symptoms (characteristic of coronary heart disease) (Table 1).

According to various studies assessing the characteristics of those who reach older ages, avoiding chronic conditions has been an essential and significant factor in an individual's survival ability [21–26]. In our study population, many individuals were observed to have lower levels of self-reported comorbidities in their older ages. Even though among the different dietary patterns there was no difference in self-reported chronic conditions (except for hypertension and high cholesterol), the reason could be due to a lower number of people with those conditions in the various diet categories, which prevented us from using regression models to better assess the associations with comorbidities. In other words, we can say that there were lower levels of chronic conditions among vegetarians in this population. Because of that, we could not accurately measure differences between diet and lifestyle factors.

Avoidance of disease seen in our cohort is comparable to other successful agers worldwide [[21], [22], [23], [24], [25], [26], [27], [28]]. It supports the implication that a factor contributing to longevity is the avoidance of chronic conditions. According to the Centers for Disease Control and Prevention (CDC), the leading causes of death in the world are chronic conditions such as heart and circulatory diseases and cancer [29]; all of those are mainly related to four lifestyle habits such as smoking, unhealthy diet, alcohol intake, and lack of exercise [30]. Our current study found that vegetarians had fewer risk factors for heart disease when compared to non-vegetarians. From the four risk factors pointed out by the CDC above [30], we found three that were explicitly lower among vegetarians: smoking, alcohol, and lack of exercise. Regarding the fourth, unhealthy diet, we saw that vegetarians had a lower BMI, less hypertension, and lower cholesterol, which are directly related to a healthy plant-based diet (Table 3).

From the plethora of past investigations and continued research on cigarette smoking, it is known that smoking is harmful to health. Currently, smoking still contributes to 80 % of lung cancers and approximately 80 % of all lung cancer deaths. It is responsible for many other cancers and can harm nearly every organ in the body [31,32]. The high prevalence of non-smokers (e.g., those who never smoked) in our population is a shared characteristic among a few long-living populations, like the Greek islanders evaluated in the MEDIS studies, where only 9–11 % were smokers [33], participants assessed in the Ikaria Study, where 17 % of men and 7 % of women were smokers [34], and those appraised by Chacón et al. [15], from the Nicoya Peninsula in Costa Rica, where 6 % of men and 0 % of women were smokers. We observed that 20.1 % of the subjects were past smokers, and 79.9 % were never smokers. Those who reported being lacto-ovo vegetarians were most likely to report never smoking, followed by vegans. Non-vegetarians were more likely to report ever smoking and less likely to report never smoking. The uniqueness of our subjects, largely being never smokers, was not observed in other studies, and this could explain the lack of differences between other risk factors in this population, as the whole cohort is primarily non-smoking, which is a very important longevity factor. As part of their lifestyle, Adventists are taught and advised to follow a holistic lifestyle that includes a vegetarian diet, exercise, non-smoking, non-drinking, etc. Therefore, the results that vegetarians are more likely to be never smokers represent the possibility that Adventists are a more health-conscious group or are more likely to maintain a healthier status and reach longevity.

The low prevalence of former smokers and the significant prevalence of never smokers has emphasized the importance of not smoking or quitting with successful aging. The researchers of the MEDIS investigation reported 9–11 % of current smokers depending on the Greek island assessed [33]. For the Ikaria Study, it was observed that 17 % of males and 7 % of females were current smokers [33,34]. However, the main difference between our investigation and other studies is the prevalence of never smokers. Observing that the majority of those who never smoked are aging successfully, that observation, along with the information offered by other researchers [15,[33], [34], [35]], is an important aspect of living successfully into older ages, whether it be due to never smoking or quitting smoking.

Another characteristic of our investigation was the evaluation of never-to-ever alcohol drinking patterns in our subjects. Within our study population, 59.3 % reported never drinking, and similarly to their smoking patterns, those who reported being lacto-ovo vegetarian were the most likely to report never drinking, followed by vegans. In reviewing other investigations assessing longevity and aging, past studies were designed to evaluate the benefits of modest drinking in the study populations [33,34,36,37].

Gmel et al. [38] concluded that subjects who reported moderate alcohol consumption were more likely to live longer compared to lifetime abstainers and ex-drinkers, which was aligned with a previously reported j-shaped relationship between alcohol consumption and mortality [39,40]. In observing the average attained age in our study population being 84.9 years of age, after excluding anyone younger than 80 from our investigation, and because a larger proportion of subjects were never drinkers, the observation of moderate drinking connected to longevity does not necessarily align with our study population. In considering this discrepant result, it is essential to note that other investigations of mortality and alcohol consumption have looked more closely at the study methodologies, and some investigators have evaluated the relationship using improved operational definitions to reassess their findings of moderate alcohol consumption being protective over life-long abstainers [[41], [42], [43]]. According to Chikritzhs, et al [41], the new methodologies and operational definitions have guided researchers toward the possibility that the assumed protective associations with low alcohol intake may be caused by confounding and bias. Moreover, another investigation on the effects of alcohol consumption was conducted by Griswold et al. [44]; the investigators evaluated the burdens attributed to alcohol and discovered that alcohol is the seventh leading cause of premature mortality and disability, and with the increase in alcohol consumption, there was an associated increase in risk for specific health outcomes, specifically for cancers, injuries, and alcohol use disorders [44]. According to the National Toxicology Program of the U.S. Department of Health and Human Services (HHS), alcohol is known to be a human carcinogen based on sufficient evidence of carcinogenicity from studies in humans [45]. Alcohol is related to at least seven types of cancer: mouth, pharynx, larynx, esophagus, female breast, liver, and colorectum [45]. The higher prevalence of study participants who are never drinkers is an uncommon feature; therefore, our study and study population do provide an indication that previously observed protective associations with alcohol consumption may be attributed to issues like confounding.

Past investigations have noted the importance of daily physical activity and have credited being physically active as a characteristic contributing to longevity and healthy aging. Many studies focused on leisure activities such as walking, gardening, or housework, as well as sports or regular exercise. Based on the researchers’ assessments of the level of physical activity and the association activity may have with longevity, they have concluded observing strong associations between being active and survival [15,33,34,37].

Compared to other studies [15,33,34,37,46,47], our population was observed to have comparable or slightly lower levels of reported physical activity. It has been noted that regular physical activity is significant in maintaining several functions as we age. Therefore, having only 19 % reported participating in regular exercise that meets recommended guidelines for activity [48] begs the question of whether being active or participating in regular exercise is a result of having better health and the ability to live to older ages, and not the cause of longevity. Another possibility related to this lower exercise level in our cohort could be related to the survey instrument asking specifically about the minutes of physical activity an individual engaged in per week. By asking for this detail, the instrument could capture the natural age-related decline in functional ability to engage in vigorous exercise, which may lead to reduced exercise and a change in physical activity patterns. Another potential cause for this low exercise response could be related to the exercise question itself. The question used for exercise asked for vigorous activity frequency and time, and expressly indicated activities that probably would not be appropriate for an 80-year-old person. Therefore, the senior participants probably did not respond to those, even though they were mostly involved in more natural exercise and something adjusted to their age, such as walking an appropriate distance. However, even though our current study cannot provide a conclusive answer to the relationship between physical activity and aging successfully, the observed similarity between our older adult population and other characteristically older populations offers suggestions about the importance of physical activity.

Our study showed that a significant number of the participants were vegetarians. For many years, researchers have explored the health effects of plant-based dietary patterns and have gained knowledge and evidence that plant-based diets are beneficial for health [49,50]. In exploring these diets, researchers have focused on inspecting traditional plant-based Mediterranean diet patterns, the vegetarian diet–characterized by abstaining from meat and meat products, or a higher total consumption of fruits and vegetables, whole grains, nuts, and legumes. Conclusions and reports from those investigations state that various dietary patterns are important for maintaining health. The investigators of different cross-sectional and prospective cohort studies have also detected that having a healthy plant-based dietary intake pattern delays the onset of chronic diseases and is considered a key feature for surviving in the oldest age groups [33,34,37,46,[50], [51], [52], [53], [54]].

In our attempts to better understand how plant-based diet patterns and survival into older ages are connected, a key feature of the Adventist population is the vegetarian dietary patterns that are optimal for evaluation of the benefits and differences attributed to various plant-based intake patterns, specifically concerning vegetarianism and the fact that many non-vegetarians consume a high intake of plant-based food. Like other cohorts that have characteristic longevity, our population has a rate of 47.3 % of non-vegetarians (See Table 2), therefore, 52.7 % of the population were vegetarians consuming a high amount of fruit and vegetables, whole grains, nuts, seeds, roots, and legumes. Regarding the individuals living in the Nicoya Peninsula, Costa Rica assessed by Chacón et al. [15], authors reported a 65 % intake of vegetables, fruit, black beans, and corn tortillas daily. In contrast, none of the participants reported daily red meat intake.

In other populations, researchers from the Iowa Centenarian Study reported that 84 % consumed two servings of beans or eggs per week and 92 % consumed two or more servings of vegetables and fruit daily [55]. In the Chinese Longitudinal Healthy Longevity Survey of older adult Chinese (≥80 years of age), 80 % reported eating beans, 90 % reported eating vegetables, with 70 % reported daily intake of vegetables [56]. From calculating adherence to the Mediterranean diet, researchers found that the study populations investigated followed more of a plant-based diet [33,34].

Finally, it is not only about living longer but also about living a high quality of life and a highly functional life that is important [57]. In our study, when we checked the variable of health perception (Table 2), we noticed that vegans have the highest percentage of excellent health perception (33.5 %). If we compare all vegetarians (29.35 %), they reported a significantly higher health perception than non-vegetarians (19.4 %). This may shed light on the quality of life for those oldest of the old in favor of a plant-based diet as we see in other long-living populations [58,59].

The ability to assess this large and diverse cohort where intake patterns, health, and lifestyle habits are varied, as well as the specific age group chosen, is a strength of our study because of the capability to generalize information and the capacity to limit confounding. Even with this strength, this investigation does have limitations. One such limitation common in cross-sectional studies is the possibility of reverse causation, whereby our subjects may have changed their dietary pattern due to the development of health issues as they aged (e.g. diabetes mellitus type II). Our study may be subject to selection bias, as individuals who chose to enroll were likely to be more health conscious. While longitudinal analyses have been conducted on the entire AHS-2 cohort, focused analyses on individuals aged 80 years and older could provide further insights by examining the relationship between diet adherence over time and corresponding health outcomes in this specific age group. Another limitation is the self-report bias. Health conditions/disease status was self-reported; therefore, we did not have medical diagnoses or biomarker levels. Again, further exploration of these biomarkers measured longitudinally with larger sample sizes will most likely produce more accurate and significant results. Moreover, caloric restriction was not examined as a factor in longevity in this study, representing a limitation. Future research should further investigate the role of caloric restriction as a potential determinant of longevity.

## 5 Conclusion

In conclusion, our AHS-2 cohort shows similar characteristics observed in the individuals who comprise the long-living cohorts worldwide. This observation provides additional evidence regarding the importance of following a plant-based diet, non-smoking, abstinence from alcohol consumption, and avoidance of disease in older ages in relation to the potential to successfully age. These features that are mostly shared among other cohorts of the oldest of the old living people around the world have indications of contributing to healthfully prolonging life with functionality, as observed by their health perception. Therefore, more investigations should be conducted to further clarify the roles of these lifestyle factors.

## CRediT authorship contribution statement

**Hildemar Dos Santos:** Writing – review & editing, Writing – original draft, Supervision, Project administration, Investigation, Funding acquisition, Conceptualization. **Alaa Alabadi-Bierman:** Writing – review & editing, Investigation. **Michael Paalani:** Writing – review & editing, Investigation. **Sen Luu Padilla:** Writing – original draft, Formal analysis. **Abel Alvarez:** Writing – review & editing, Investigation. **W. Lawrence Beeson:** Writing – review & editing, Validation, Methodology, Formal analysis, Data curation. **Gary E. Fraser:** Supervision, Software, Resources, Methodology, Investigation, Funding acquisition.

## Declaration of competing interest

The author(s) declared no potential conflicts of interest concerning this article's research, authorship, and publication.
